# Identification and Validation of Prognostic Markers for Lung Squamous Cell Carcinoma Associated with Chronic Obstructive Pulmonary Disease

**DOI:** 10.1155/2022/4254195

**Published:** 2022-08-17

**Authors:** Zheng Li, Dan Xu, Jing Jing, Jing Wang, Min Jiang, Fengsen Li

**Affiliations:** ^1^The Traditional Chinese Medicine Hospital Affiliated with Xinjiang Medical University, Urumqi, China; ^2^National Clinical Research Base of Traditional Chinese Medicine, Urumqi, China; ^3^Key Laboratory of Xinjiang Uygur Autonomous Region(respiratory Disease), Urumqi, China

## Abstract

**Background:**

Globally, the incidence and associated mortality of chronic obstructive pulmonary disease (COPD) and lung carcinoma are showing a worsening trend. There is increasing evidence that COPD is an independent risk factor for the occurrence and progression of lung carcinoma. This study aimed to identify and validate the gene signatures associated with COPD, which may serve as potential new biomarkers for the prediction of prognosis in patients with lung carcinoma.

**Methods:**

A total of 111 COPD patient samples and 40 control samples were obtained from the GSE76925 cohort, and a total of 4933 genes were included in the study. The weighted gene coexpression network analysis (WGCNA) was performed to identify the modular genes that were significantly associated with COPD. The KEGG pathway and GO functional enrichment analyses were also performed. The RNAseq and clinicopathological data of 490 lung squamous cell carcinoma patients were obtained from the TCGA database. Further, univariate Cox regression and Lasso analyses were performed to screen for marker genes and construct a survival analysis model. Finally, the Human Protein Atlas (HPA) database was used to assess the gene expression in normal and tumor tissues of the lungs.

**Results:**

A 6-gene signature (DVL1, MRPL4, NRTN, NSUN3, RPH3A, and SNX32) was identified based on the Cox proportional risk analysis to construct the prognostic RiskScore survival model associated with COPD. Kaplan–Meier survival analysis indicated that the model could significantly differentiate between the prognoses of patients with lung carcinoma, wherein higher RiskScore samples were associated with a worse prognosis. Additionally, the model had a good predictive performance and reliability, as indicated by a high AUC, and these were validated in both internal and external sets. The 6-gene signature had a good predictive ability across clinical signs and could be considered an independent factor of prognostic risk. Finally, the protein expressions of the six genes were analyzed based on the HPA database. The expressions of DVL1, MRPL4, and NSUN3 were relatively higher, while that of RPH3A was relatively lower in the tumor tissues. The expression of SNX32 was high in both the tumor and paracarcinoma tissues. Results of the analyses using TCGA and GSE31446 databases were consistent with the expressions reported in the HPA database.

**Conclusion:**

Novel COPD-associated gene markers for lung carcinoma were identified and validated in this study. The genes may be considered potential biomarkers to evaluate the prognostic risk of patients with lung carcinoma. Furthermore, some of these genes may have implications as new therapeutic targets and can be used to guide clinical applications.

## 1. Introduction

Chronic obstructive pulmonary disease (COPD) and lung carcinoma are becoming major public health concerns due to increased morbidity and mortality. COPD causes persistent respiratory symptoms and progressive airflow obstruction and affects more than 200 million people worldwide [1, 2]. Aging and smoking contribute to the mortality of approximately 400,000 individuals each year due to COPD, making it the third leading cause of death worldwide [3–5]. COPD is a risk factor for lung carcinoma, and the malignancy contributes to the majority of cancer-related deaths globally, causing more than 1.3 million deaths annually [6, 7]. The 5-year survival rate of patients with advanced lung carcinoma is only 15% [8]. In the United States, squamous cell carcinoma (SCC) accounts for approximately 20% of all lung carcinomas and is the predominant histological type of malignancy in men [9]. Recent epidemiological studies have demonstrated the association between the progression of COPD and lung carcinoma, wherein patients diagnosed with lung carcinoma were at a higher risk of COPD (up to 4.5 fold) compared to those with cancer alone. Additionally, the occurrence of lung carcinoma was reported to be associated with severe COPD [10]. The poor prognosis of lung carcinoma may be associated with reduced lung function in patients with COPD; higher grades of COPD are associated with increased rates of postoperative pulmonary complications, reduced long-term survival, and higher cancer-related mortality [11]. Pulmonary complications after lung carcinoma surgery are a major cause of morbidity in patients with COPD [12]. Therefore, assessment of the underlying mechanisms of COPD and lung carcinoma is crucial for the development of novel therapeutic strategies.

Lung carcinoma and COPD have some common features; therefore, they are closely associated [13–16]. Recent studies have shown that COPD is a chronic inflammatory condition of the lungs, which could be associated with potential genetic errors, causes recurrent damage and repair, stimulates cell renewal, and eventually induces lung carcinoma. Increased COPD-induced oxidative stress could be a key mechanism influencing the prognosis and survival of patients with lung carcinoma by causing DNA damage and affecting the oncogenic DNA repair capacity [17]. Smoking is responsible for both COPD and lung carcinoma as the components of smoke induce inflammation and oxidative stress in the lung tissue, which alters the transcription and activation of protein hydrolases and their inhibitors. This leads to an imbalance in the lung parenchyma and causes lung tissue damage and a higher predisposition to lung carcinoma [18]. SCC is more closely associated with smoking than any other nonsmall cell lung carcinoma (NSCLC) [19]. A history of smoking and COPD are important risk factors for lung squamous cell carcinoma (LUSC) [20]. The presence of COPD is known to increase the risk of the squamous cell histological subtype by at least four times [21]. In addition to environmental smoke exposure, abnormal inflammatory and immune responses are associated with the progression of both COPD and SCC [22].

Currently, there are no known biomarkers with high sensitivity and specificity for the detection and prognostication of LUSC in patients with COPD or a heavy smoking habit. Evaluation of the biological pathways and networks associated with COPD and LUSC is important for the screening, prevention, diagnosis, and treatment of patients. In the present study, we identified a 6-gene signature prognostic risk evaluation model based on TCGA and GEO cohorts using a bioinformatics approach. The model was validated using internal and external validation sets, and the results indicated its stability and reliability in predicting the prognosis of patients with LUSC. Finally, the Human Protein Atlas (HPA) database was used to evaluate the gene expression in normal and tumor tissues of the lungs. The findings of the present study may have implications for considering novel biomarkers to predict the prognosis of patients with LUSC and identify the therapeutic targets.

## 2. Materials and Methods

### 2.1. Preprocessing of Transcriptome Data

Microarray expression data of COPD patients were obtained from the GEO database. The GSE76925 cohort was selected, which consisted of 151 samples, including 111 patient samples and 40 control samples that were used for subsequent analysis. The microarray cohort was Illumina HumanHT-12 V4.0. Genes that did not localize onto a chromosomal location were excluded. If multiple probes corresponded to a gene, the average expression was considered for that gene. A total of 4933 genes were included in the study. RNAseq (in TPM) and clinicopathological data of LUSC were obtained from the TCGA database (TCGA-LUSC cohort). Samples where the ‘time to live' was unknown were excluded, and finally, 490 patients were selected for the analysis (Table 1).

### 2.2. Construction of the Coexpression Network

The WGCNA was performed based on sample grouping, and all genes were included. Genes in the top 75% of the median absolute deviation were included in the preprocessing stage and genes with values greater than 0.01 were selected for the analysis.

A sample dendrogram and trait heatmap were visualized using the WGCNA package. Firstly, the outliers in the samples were checked and removed. Subsequently, the power of *β* value was introduced to transform the similarity matrix into an adjacency matrix. In this study, we defined the adjacency matrix using soft thresholding with beta = 3 (scale-free R2 selection = 0.86). On this basis, we constructed a scale‐free network and topological overlap matrix (TOM). Ultimately, genes with highly absolute correlations were clustered into the same module to generate a cluster dendrogram by The Dynamic Tree Cut method. The minimum number of genes per module was preset to 10.

### 2.3. Screening of Clinically Significant Modules

Based on the WGCNA, the genes were classified into different modules to identify the association between the modules and clinical features. Module significance (MS) was defined as the correlation between the major components of a gene module and the clinical features. MS was calculated using the average GS value of all genes in a module to prioritize those significantly associated with COPD. Three modules with the largest absolute values were found to be closely associated with the clinical features; therefore, these modules and the clinical features were selected for further analysis.

### 2.4. Machine Learning-Based Variable Screening and Model Construction

TCGA-LUSC cohort was obtained, and candidate genes were extracted from the module. Univariate Cox regression analysis was performed to select the significant candidate genes (*p* < 0.05), followed by the Lasso-Cox regression analysis. Finally, a multivariate Cox regression analysis was performed to prioritize the significant genes (*p* < 0.05) as the markers. Multivariate Cox regression analysis based on the marker genes was performed to construct a survival analysis model. The RiskScore was calculated as follows:

(1) $$\begin{array}{c} \text{Risk Score}=\sum_{i=1}^{N}\exp{\;}^{*}\text{coef}, \end{array}$$

where *N* is the number of genes, exp is the expression value of the gene and coef.

### 2.5. Protein Expression Analysis

The HPA provides information regarding the tissue and cellular distribution of 26,000 human proteins, wherein the protein expression in the cell lines of normal and tumor tissues is evaluated using specific antibodies. This database was used to evaluate the gene expression in normal and tumor tissues of the lungs.

## 3. Results

### 3.1. Identification of the COPD-Associated Modules

**Figure 1:**
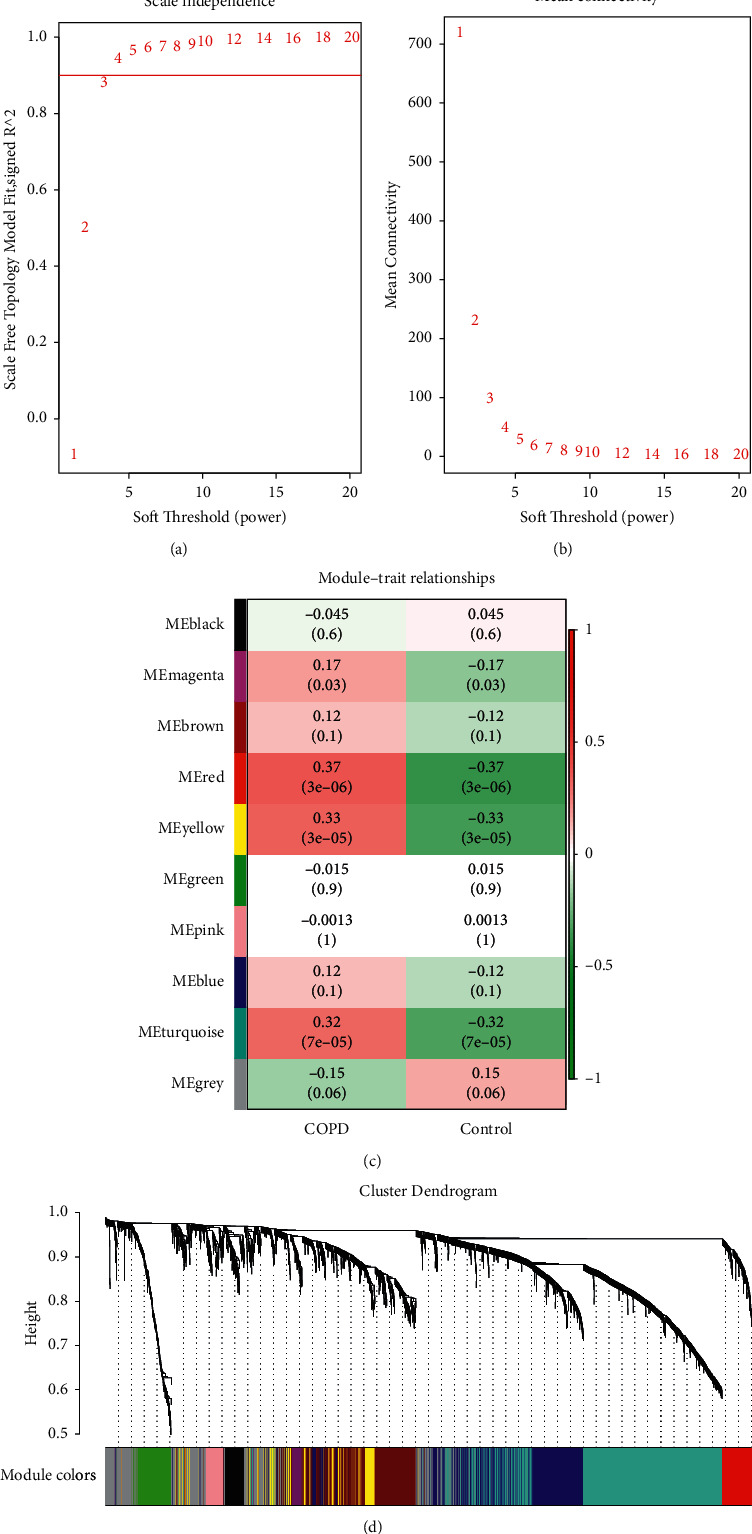
Identification of the modules associated with the clinical features of COPD. (a) Analysis of the scale-free fit index for various soft-thresholding powers (*β*). (b) Analysis of the mean connectivity for various soft-thresholding powers. (c) Heatmap of the correlation between module eigengenes and clinical traits of COPD. (d) Dendrogram based on a dissimilarity measure (1-TOM).

A total of 3699 genes were selected in this study. The WGCNA package was used for the mRNA coexpression network analysis, in which the optimal *β* value of 3 was determined by picking the *β* value. The scale-free network was validated for *β* = 3 (Figure 1(a) and 1(b)). Finally, a total of ten modules were identified. Modules associated with COPD were evaluated, and those with larger MS were considered to have a greater correlation with disease progression (Figures 1(c) and 1(d)). The ME in the red, yellow, and turquoise modules were clinically more significant for disease progression than any other disease module and were selected for further analysis. The three aforementioned modules included a total of 1535 genes.

### 3.2. Functional Annotation and Analysis

**Figure 2:**
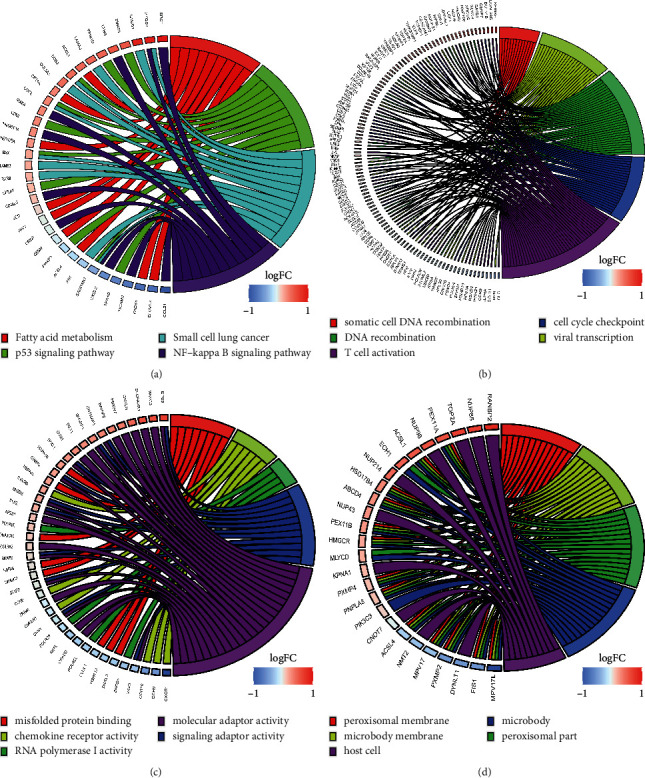
Enriched biological functions and pathways associated with the module genes. (a) Gene enrichment in tumor-associated pathways. (b) Gene enrichment in biological processes. (c) Gene enrichment in molecular functions. (d) Gene enrichment in cellular components.

Functional annotation, KEGG pathway, and GO functional enrichment analyses were performed on the three modules of genes closely associated with COPD. The results indicated that these genes were significantly enriched in fatty acid metabolism, p53 signaling, NF-kappa B signaling, and other tumor-related pathways (Figure 2(a)). Tumor pathways involved in COPD-related genes regulate the occurrence and development of lung cancer to a certain extent.

Additionally, the genes were enriched in somatic cell DNA recombination, DNA recombination, T-cell activation, cell cycle checkpoint, and other biological functions (Figure 2(b)). Furthermore, the analyses revealed that the genes were enriched in molecular functional categories such as misfolded protein binding and chemokine receptor activity (Figure 2(c)), as well as in cellular components such as the microbody membrane and peroxisomal membrane (Figure 2(d)).

### 3.3. Identification of the Important Genes and Model Construction

**Figure 3:**
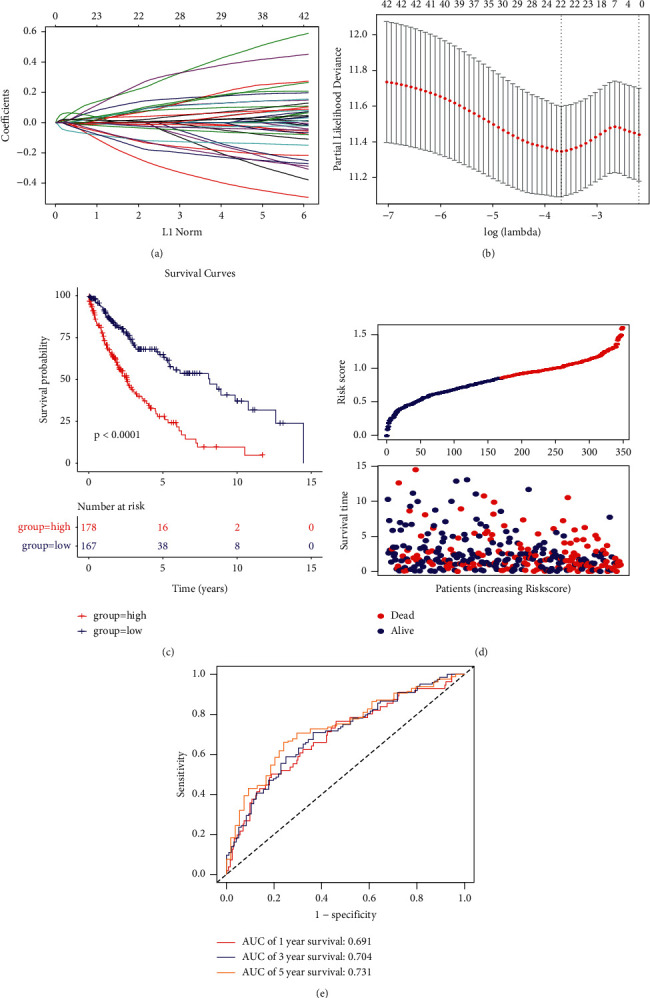
Selection of the optimal tuning parameter lambda using the Lasso model. (a) Trajectory of each independent variable; the horizontal axis represents the log value of the independent variable lambda, and the vertical axis represents the coefficient of the independent variable. (b) Confidence intervals for each lambda. (c) Distribution of the Kaplan–Meier survival curves of the gene signature in the training set. (d) RiskScore, time to live, survival status, and gene signature expression in TCGA training set. (e) ROC curves and AUC of the gene signature classification.

The expression data associated with LUSC was obtained from TCGA and the sample data set was randomly split in the ratio of 7 : 3. In the training data (*N* = 245), the intersection sets were taken for candidate genes with TCGA genes, and a total of 1172 genes were found to overlap. Univariate Cox regression analysis was performed on the 1172 genes, following which 42 candidate genes were selected. Subsequently, the Lasso-Cox regression analysis was performed on these genes and 22 important genes were selected. The Lasso-Cox regression analysis was performed using the R package glmnet and the trajectories of each independent variable were analyzed as shown in Figure 3(a). Subsequently, a model was constructed using 5-fold cross-validation to determine the confidence intervals at each lambda, as shown in Figure 3(b). Our results indicated that the model was optimal at lambda = 0.025. For this reason, the 22 genes at lambda = 0.025 were selected as the target genes for the next step. Further, multivariate Cox regression analysis was performed on these 22 genes. Finally, six marker genes (DVL1, MRPL4, NRTN, NSUN3, RPH3A, and SNX32) were selected (*p* < 0.05). In addition, multivariate Cox regression analysis was performed to determine the correlation coefficients of the marker genes, as well as the associated prognostic expression using the linear combination as shown below:

(2) $$\begin{array}{c} \text{Risk Score}=DVL1{\;}^{*}0.5106+MRPL40{\;}^{*}-0.4385+NRTN{\;}^{*}-0.2223+NSUN3{\;}^{*}0.2907+RPH3A{\;}^{*}0.1063+SNX32{\;}^{*}-0.2426. \end{array}$$

The RiskScore for each sample was calculated separately based on the gene expression in the samples. Samples with values greater than and lesser than zero were classified into the high-risk and low-risk groups, respectively, based on the median value of the RiskScore. In addition, Kaplan–Meier (KM) curves were plotted, as shown in Figure 3(c). The distribution of the RiskScore in the samples is shown in Figure 3(d), which indicates that the proportion of deaths in the samples with high scores was significantly greater than in those with low scores, suggesting a worse prognosis in patients with a high RiskScore. ROC analysis of the prognostic classification of the RiskScore was also performed using the R package timeROC, wherein the classification efficiency of the prognostication was analyzed at 1, 3, and 5 years. As shown in Figure 3(e), the model demonstrated a high AUC.

### 3.4. Robustness of the Model across Different Platforms

**Figure 4:**
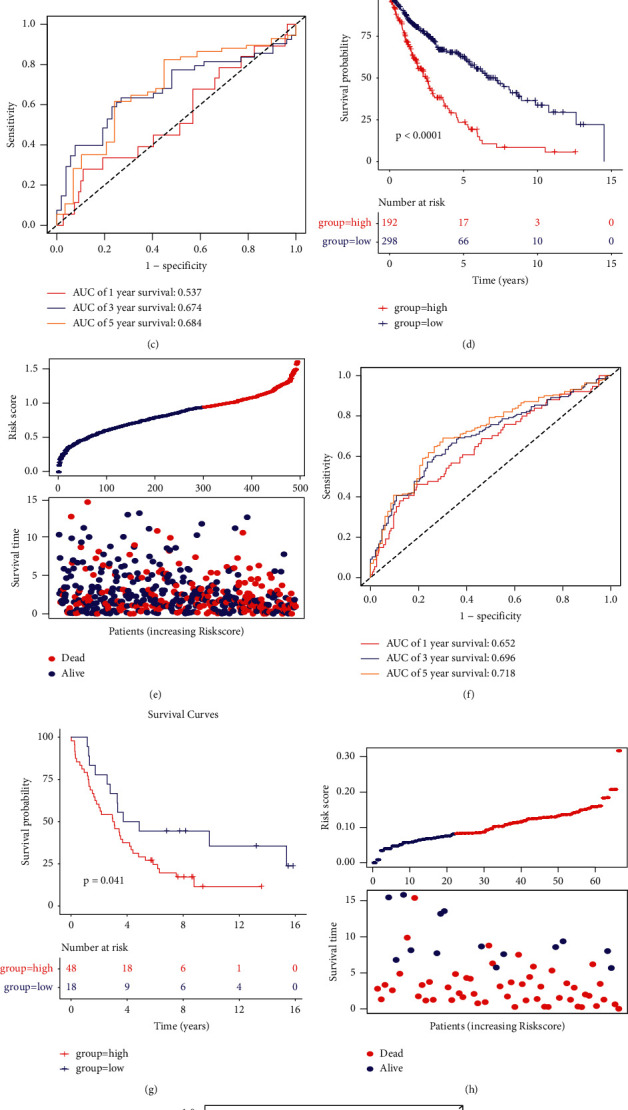
RiskScore performance in the different cohorts. (a) Distribution of the Kaplan–Meier (KM) survival curves of the gene signature in the test set. (b) RiskScore, time to live (TTL), survival status, and gene signature expression in the test set. (c) ROC curves and AUC of the gene signature in the test set. (d) Distribution of the KM survival curves of the gene signature in the full set. (g) Distribution of the KM survival curves of the gene signature in the GSE37745 cohort; (h) RiskScore, TTL, survival status, and gene signature expression in the GSE37745 cohort; (i) ROC curves and AUC of the model in the GSE37745 cohort.

Parameters of the training set were used in the TCGA validation set and the full data set to determine the robustness of the model. The RiskScore of each sample was calculated separately based on the gene expression levels. Using the analysis described above, the KM curves were plotted as shown in Figures 4(a) and 4(d). Our results indicated that the model could significantly differentiate between the prognoses of patients in both the test and full sets. The RiskScore distribution of the samples in the test and full sets are shown in Figures 4(b) and 4(e). The results confirmed that the samples with a high RiskScore had a worse prognosis. ROC analysis of the prognostic classification of the RiskScore was performed, as described previously. Figures 4(c) and 4(f) demonstrate that the model has a high AUC in both the test and full sets. Validation of the model was performed in the external independent cohort, GSE37745, using the same approach as described above. The results showed that the model could significantly differentiate between the prognoses of patients (Figure 4(g)). A high RiskScore sample was associated with a worse prognosis (Figure 4(h)), and the model achieved the predicted ROC of 0.73 at 5 years (Figure 4(i)).

### 3.5. Evaluation of the Prognosis Prediction Ability of the Model in Different Clinical Indicators

**Figure 5:**
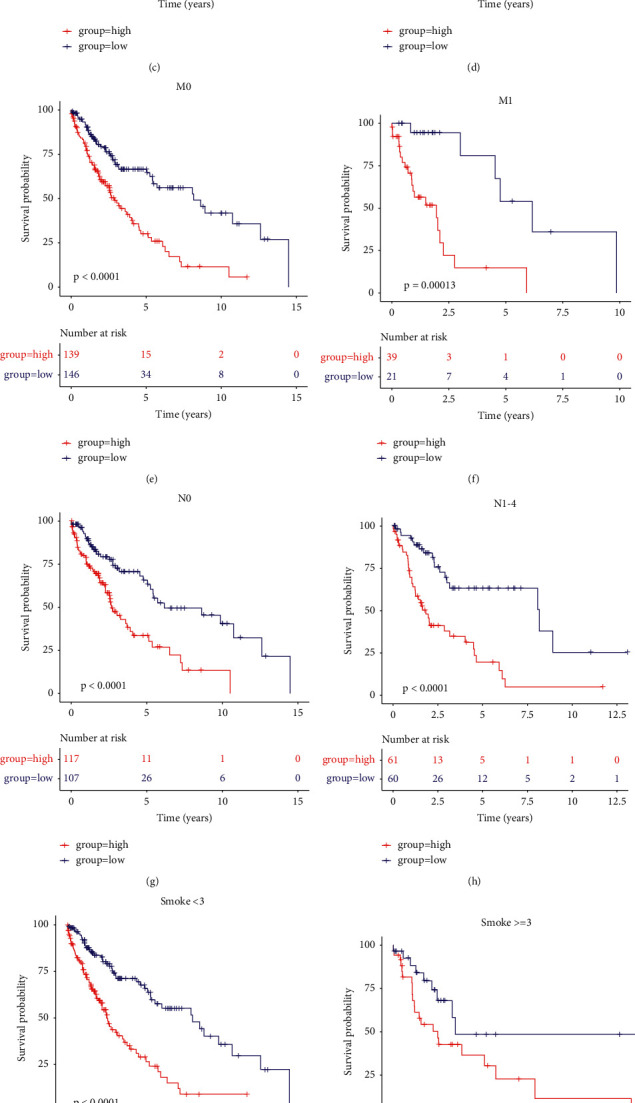
The RiskScore performance in the training cohort with clinical indicators (A–L): The survival plot of OS with clinical variables including age, sex, M-stage, N-stage, T-stage, and stage.

To assess the prognostic, predictive power of RiskScore in clinical subgroups, we performed a survival analysis of subgroups. Age, gender, stage N, stage *M*, smoking habits, and clinical stage were the indicators selected for evaluation. It demonstrated that the model indicated significant survival differences in age, gender, N-stage, M -stage, smoking habit, and clinical stage indicators (Figures 5(a)–5(l)). However, it should be noted that RiskScore was not significant in predicting patient prognosis when age was less than or equal to 60 years.

### 3.6. Validation of the Independent Prognostic Efficacy of the Model

**Figure 6:**
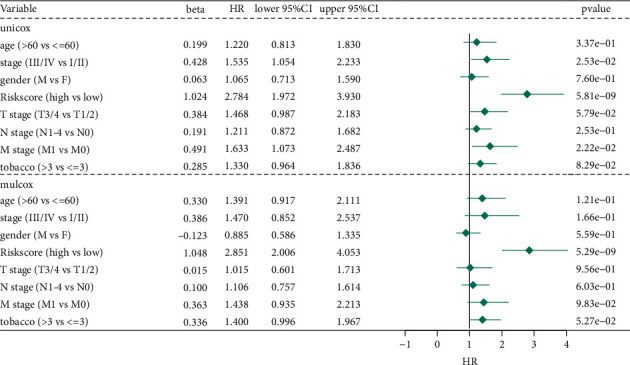
Univariate and multivariate Cox regression analyses for OS in TCGA training cohort.

Univariate and multivariate Cox regression analyses were performed to investigate the independent prognostic efficacy of the model considering the sex, age, tumor stage, and RiskScore of the patients. In TCGA cohort, univariate Cox regression analysis revealed that the RiskScore, M-stage, and stage were significantly associated with the prognosis, while the corresponding multivariate Cox regression analysis indicated that the RiskScore (HR = 2.85, 95% CI = 2.006–4.053, *p* < 1*e* − 55) was significantly associated with survival. The results suggested that the model proposed in this study was an independent risk factor for the prognosis of patients with lung carcinoma. In addition, the prognosis was significantly associated with the number of cigarettes smoked, wherein patients who smoked more than three cigarettes had a worse prognosis than those who smoked less than three cigarettes (insert *p*-value) (Figure 6).

### 3.7. Nomogram Construction

**Figure 7:**
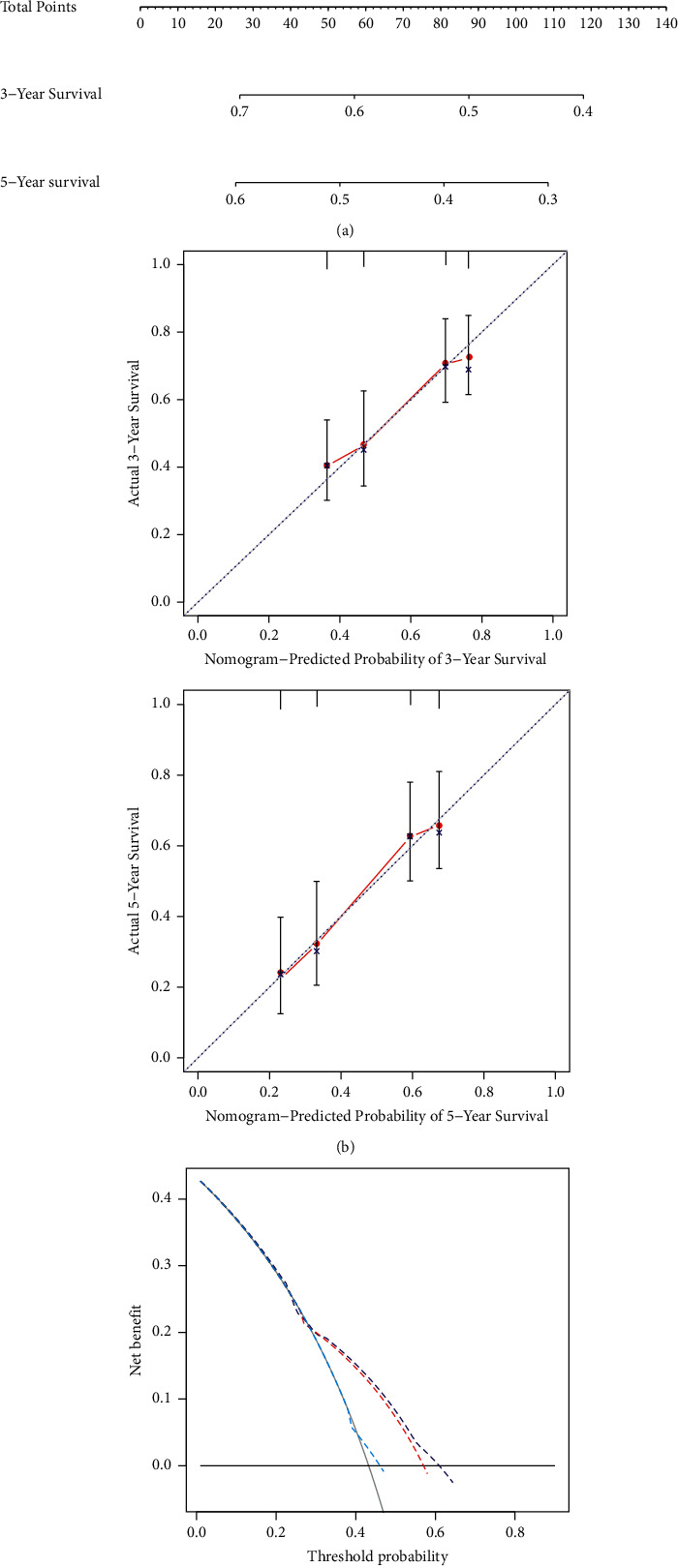
(a) Nomogram predicting the 3-year and 5-year survival. (b) Calibration curves for the 3-year and 5-year survival. The curves depict the calibration of each model based on the agreement between the predicted probabilities and observed outcomes in the training set. (c) Decision curve analysis of the nomogram for 3-year and 5-year survival.

A nomogram is a visually effective representation of the results of risk models and is convenient for predicting the outcomes. The length of a straight line in a nomogram indicates the impact of different variables and their effect on the outcome. Based on the results of the multivariate analysis, the clinical features including the RiskScore and the number of cigarettes smoked, were used to construct a nomogram (Figure 7(a)). It showed that the RiskScore had the greatest impact on survival prediction, indicating that the 6-gene-based risk model could predict the prognosis better. In addition, the prediction accuracy of the model was evaluated using the calibration curve, as shown in Figure 7(b). The prediction calibration curves at 3 years and 5 years nearly coincided with the standard curve, which suggested that the model had good accuracy. Furthermore, decision curve analysis (DCA) revealed that the benefits of the RiskScore and nomogram were significantly higher than the extreme curves (Figure 7(c)). Our results indicate that the RiskScore and nomogram have potential clinical applicability.

### 3.8. Correlation between Core Gene Expression and Pathways

**Figure 8:**
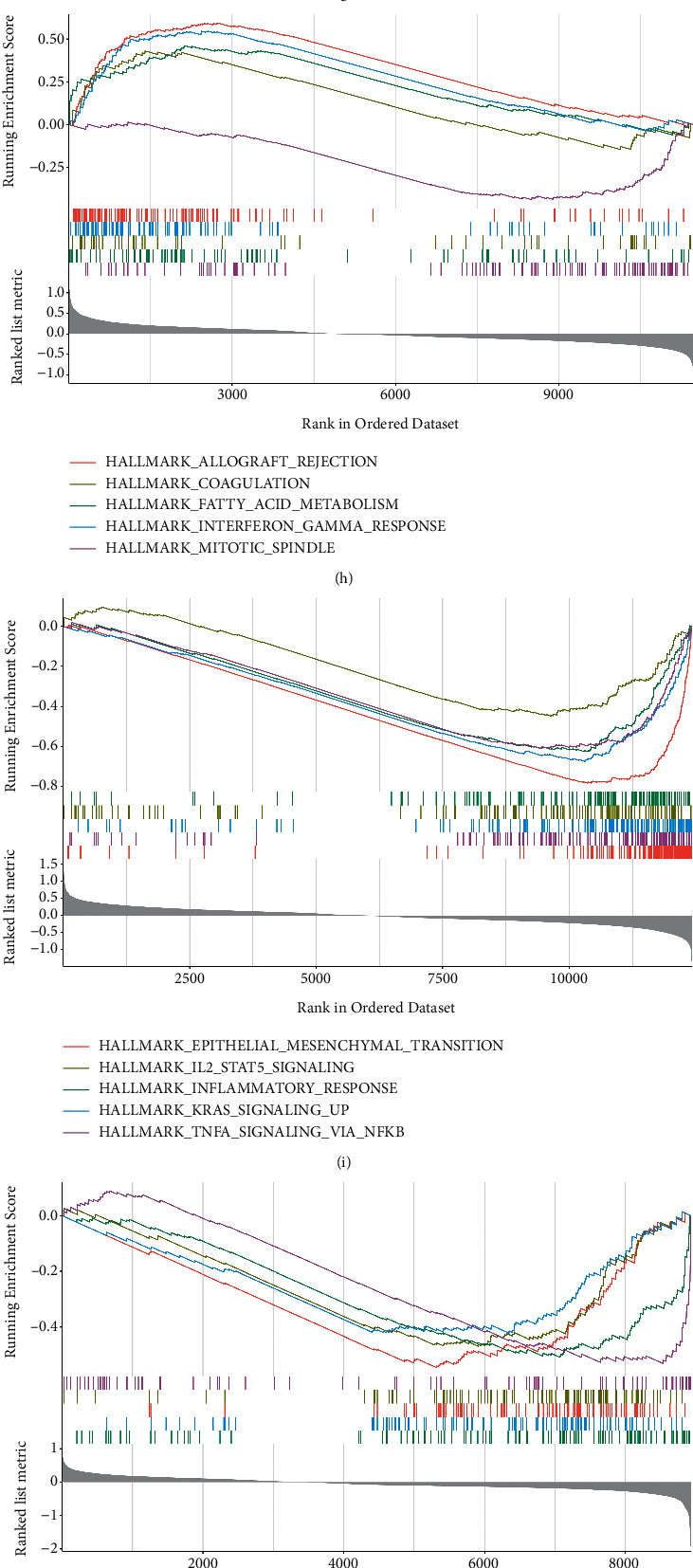
Correlation between the expression and pathways of the core genes. (a–e) Gene expression in the HPA database. (f) Differences in the expression of the six genes in the GSE31446 cohort. (g) Differences in the expression of the six genes in TCGA cohort. (h–m) GSEA of the six genes.

The protein expressions of the six genes were analyzed based on the HPA database. The NRTN gene was not expressed in the HPA database. As shown in Figure 8, the expression of DVL1, MRPL4, and NSUN3 was higher in tumors than in normal tissues, while that of RPH3A was lower. SNX32 was highly expressed in both the tumor and paracarcinoma tissues (Figures 8(a)–8(e)). The gene expression profiles of LUSC were obtained from TCGA and GEO (GSE31446) databases. The differences in expression of the six genes in the two cohorts were analyzed separately. As shown in Figure 8, MRPL4, NRTN, and NSUN3 were significantly upregulated in both TCGA (Figure 8(g)) and GEO (Figure 8(f)) cohorts, while the expression of RPH3A was significantly downregulated. These observations were consistent with the expressions reported in the HPA database.

Based on the median expression levels of the genes, TCGA-LUSC samples were classified into high- and low-risk groups. GSEA was performed for each gene using the clusterProfilter package, and the top five pathways that were significantly enriched were considered for representation. The results showed that the DVL1 gene was involved in HALLMARK_FATTY_ACID_METABOLISM and the MRPL4 gene in the HALLMARK_EPITHELIAL_MESENCHYMAL_TRANSITION, HALLMARK_KRAS_SIGNALING_UP, and HALLMARK_IL2_STAT5_ SIGNALING pathways. Likewise, the NRTN and RPH3A genes were involved in the HALLMARK_KRAS_SIGNALING, the NSUN3 gene in the HALLMARK_G2M_CHECKPOINT, and the SNX32 gene in HALLMARK_REACTIVE_OXYGEN_SPECIES pathways (Figures 8(h)–8(m)).

## 4. Discussion

Globally, COPD and lung carcinoma are major causes of death. A history of smoking and the presence of COPD are important risk factors for LUSC. In particular, the presence of COPD is known to increase the risk of the squamous cell histological subtype by more than four times [21]. Understanding the association between COPD and LUSC is important for the treatment and prognosis of patients. In this study, a comprehensive evaluation was performed to identify and validate a 6-gene prognostic signature. Validations were performed on the internal and overall test sets, as well as on an external set. We speculated that risk modeling to assess the potential association between the RiskScore and the survival rate of patients with LUSC might provide a better understanding of the common underlying mechanisms of COPD and LUSC, as well as potential new biomarkers to determine the prognosis of patients with LUSC.

In this study, the WGCNA was performed to select COPD-related modules from a total of 1535 genes. The KEGG pathway and GO functional enrichment analyses were performed on the genes in the three modules. In addition, the expression data of LUSC were obtained from TCGA and were randomly classified into the training and validation sets in the ratio of 7 : 3. Univariate Cox regression and Lasso-Cox regression analyses were performed to construct a RiskScore prognostic model consisting of the six marker genes (DVL1, MRPL4, NRTN, NSUN3, RPH3A, and SNX32). The results suggested that high RiskScore samples had a worse prognosis. Validation in different cohorts showed good robustness of the model. Evaluation of the nomogram model based on the RiskScore using calibration curves demonstrated good accuracy, and the DCA indicated the potential clinical applicability of the model. We evaluated the merits of the RiskScore based on different clinical indicators by prognostic analysis of the risk model and clinical features. The results indicated that the 6-gene signature model significantly distinguished between high- and low-risk groups based on age (>60 years), sex, M-stage, N-stage, smoking habit, and stage. Thus, the proposed model demonstrated good predictive ability for different clinical factors as well. The multivariate Cox regression analysis indicated that the constructed risk model could be considered an independent prognostic risk factor.

The protein expressions of the six genes were analyzed in the HPA database, wherein the NRTN gene was not expressed; DVL1, MRPL4, and NSUN3 were relatively high in tumor tissues, RPH3A was relatively lower in tumors, and SNX32 was highly expressed in both the tumor and paracarcinoma tissues. The gene expression profile of LUSC was obtained from TCGA and GEO (GSE31446) databases, and the differences in expression of the six genes were analyzed separately in the two cohorts. The results revealed that the MRPL4, NRTN, and NSUN3 genes were significantly upregulated in both TCGA and GEO cohorts, while the RPH3A gene was significantly downregulated in tumor samples, consistent with that reported in the HPA database and previous studies (insert citation of the previous studies). The DVL gene plays a central role in Wnt signaling, including the canonical and noncanonical Wnt signaling pathways that control several cellular processes such as cell proliferation, survival, migration, differentiation, polarity, and stem cell renewal [23–25]. Three homologous genes (DVL1, DVL2, and DVL3) with high similarity have been identified in humans [26]. Overexpression of DVL is known to enhance the activation of Wnt signaling and may play a role in the progression of several cancers [27–37]. The proto-oncogene DVLl is an important component of the Wnt/*β*-catenin signaling pathway and is a key cytoplasmic regulator that prevents degradation of the *β*-catenin protein, and its high expression in tumors is associated with malignancy [38]. Previous studies have reported that both DVL1 and DVL3 proteins are overexpressed in NSCLC and are associated with a poor prognosis. DVL1 affects the biological behavior of lung carcinoma cells primarily through the *β*-catenin (canonical Wnt) pathway, while DVL3 acts primarily through the p38 and JNK pathways [28]. Amplification and increased expression of the DVL1 gene may have implications in human breast cancer through disruption of the Wnt signaling pathway [30]. Evidence suggests that overexpression of the DVL1 gene is closely associated with liver metastasis in rectal cancer [33]. Additionally, amplified expression of the DVL1 gene may be important in the progression of human cervical SCC through disruption of the Wnt signaling pathway [34]. Taken together, based on their roles in physiological and pathophysiological processes, this protein family can be considered a potential target for cancer therapy. Glial cell line-derived neurotrophic factor (GDNF) binds to the GDNF family receptor, GFR*α*2, and regulates the response to peripheral stimuli by activating RET tyrosine kinase [39, 40]. There are limited studies regarding the NRTN gene in oncology. However, some studies have reported that this gene is specifically upregulated in pancreatic cancer, wherein it contributes to the sustained proliferation and increased aggressiveness of the malignancy [41]. Additionally, RET, a receptor for GDNF and a neurotrophic factor (NTN), is a transmembrane tyrosine kinase that transduces RET-mediated signals in a variety of signaling pathways, in particular, the Ras signaling and the phosphatidylinositol-3 kinase pathways [42]. The RET fusion gene is a novel oncogene identified recently in NSCLC, and young Asian women, nonsmokers, and patients with lung adenocarcinoma are good candidates for personalized diagnosis and treatment [43]. The MRPL4 gene is located on chromosome and encodes for a protein with 319 amino acids [44]. Some studies have indicated that the MRPL4 gene can be a potential therapeutic target for the treatment of hypertension and stroke [45]. This gene has been reported to have a strong correlation with the susceptibility to allergic rhinitis [46]. The RNA cytosine methyltransferase NSUN3 produces 5-methylcytosine in the anticodon loop of the mitochondrial tRNA^Met^ and regulates embryonic stem cell differentiation by promoting mitochondrial activity. Deletion of functional mutations in NSUN3 is associated with multisystem mitochondrial diseases such as early-onset mitochondrial encephalomyopathy and epilepsy [47–49]. RPH3A is a synaptic vesicle-associated protein that is involved in the regulation of exocytosis at presynaptic sites and plays an important role in synaptic stabilization. This protein can be a potential new target for the treatment of levodopa-induced dyskinesia [50–52]. The SNX32 gene is associated with an increased risk of Alzheimer's disease [53]. There are limited studies on the MRPL4, NSUN3, RPH3A, and SNX32 genes in oncology. Patients with LUSC (TCGA) were divided into high- and low-risk groups according to their median gene expression levels, and GSEA was performed for each gene. Our results showed that the DVL1 gene was involved in HALLMARK_FATTY_ACID_METABOLISM and the MRPL4 gene in HALLMARK_EPITHELIAL_MESENCHYMAL_TRANSITION, HALLMARK_KRAS_SIGNALING_UP, and HALLMARK_IL2_STAT5_ SIGNALING pathways. Likewise, the NRTN and RPH3A genes were involved in the HALLMARK_KRAS_SIGNALING, NSUN3 in the HALLMARK_G2M_CHECKPOINT, and the SNX32 gene in the HALLMARK_REACTIVE_OXYGEN_SPECIES pathways. The majority of these pathways are associated with tumor progression. Among them, the G2M checkpoint pathway is an important component of the cell cycle. Some studies have shown that high G2M signaling pathway scores are associated with cell proliferation and worse survival in pancreatic cancer patients [54]. Similarly, cases of breast cancer demonstrating high activity of the G2M pathway genes are more aggressive and more likely to metastasize [55]. Epithelial-mesenchymal transition (EMT) confers metastatic properties to cancer cells [56, 57], and studies have described the contribution of the transition to the progression of lung carcinoma, cancer stem cells, and acquisition of resistance to EGFR tyrosine kinase inhibitors and chemotherapy [58]. The reactive oxygen species-mediated signaling pathway further activates proto-oncogene signaling pathways and plays an important role in tumorigenesis and progression. Therefore, antioxidant inhibitors could be promising candidates for anticancer therapy [59]. The results of the present study might be useful for clinicians to assess the prognosis of patients with lung carcinoma and select the appropriate therapeutic targets.

However, this study has certain limitations. In this retrospective study, we have focused only on the microarray expression cohort with a small sample size, which could be associated with selection bias. In addition, some important genes may have been missed during the multistep selection process, thereby limiting the utilization of the RiskScore. Future investigations are required to validate further this risk model in clinical settings, assess the prediction accuracy, and enhance clinical applications. In addition, future studies should be conducted on larger sample sizes and include cellular level and animal-based experimental validations to elucidate the mechanism of action of these targets.

## 5. Conclusions

In conclusion, we identified a 6-gene signature prognostic risk evaluation model constructed using a bioinformatics approach based on the cohorts from TCGA and GEO databases. We validated the stability of the model and its reliability in predicting the prognosis of patients with lung carcinoma. The present study increases our understanding of the relationship between lung carcinoma and COPD. The genetic markers may serve as promising prognostic biomarkers and may have implications as potential therapeutic targets for patients with lung carcinoma.

## Figures and Tables

**Table 1:** Sample data table.

Clinical features	TCGA-train	TCGA-test	TCGA-all
*OS*			
0	193	87	280
1	152	58	210

*T stage*			
T1	80	32	112
T2	201	84	285
T3	49	21	70
T4	15	8	23

*N-stage*			
N0	224	90	314
N1	83	43	126
N2	29	11	40
N3	4	1	5
NX	5	0	5

*M stage*			
M0	285	117	402
M1	6	1	7
MX	50	27	77
NA	4	0	4

*Stage*			
I	173	66	239
II	104	53	157
III	59	24	83
IV	6	1	7
NA	3	1	4

*Sex*			
Male	267	96	363
Female	78	49	127

*Age (years)*			
≤60	80	26	106
>60	261	118	379
NA	4	1	5

*Cigarette smoking (n)*			
≤3	167	65	232
>3	171	75	246
NA	7	5	12

## Data Availability

We have provided detailed information about the materials and methods in our manuscript.

## Abbreviations

COPD: Chronic obstructive pulmonary disease
HPA: Human Protein Atlas
SCC: Squamous cell carcinoma
NSCLC: Non-small cell lung carcinoma
LUSC: Lung squamous cell carcinoma
MS: Module significance
DCA: Decision curve analysis.
